# Why are men taller than women?: Genetic and hormonal factors involved in sex differences in human height

**DOI:** 10.1038/s10038-025-01384-4

**Published:** 2025-08-14

**Authors:** Maki Fukami, Tsutomu Ogata, Atsushi Hattori

**Affiliations:** 1 Department of Molecular Endocrinology, National Research Institute for Child Health and Development, Tokyo, Japan; 2 Department of Pediatrics, Hamamatsu University School of Medicine, Hamamatsu, Japan; 3 Department of Pediatrics, Hamamatsu Medical Center, Hamamatsu, Japan

**Keywords:** Development, Genetics research

**TO THE EDITOR:**

On average, adult men are ~13 cm taller than women [1]. Male-dominant skeletal growth in humans is primarily ascribed to sex hormones, particularly testicular androgens, and male-specific genetic factors [1]. The sex-biased expression of autosomal genes likely accounts for only ~1.6 cm of this difference [2]. Still, all attempts to identify a male-specific growth-controlling gene on the Y chromosome (GCY) have failed. No gene on the human Y chromosome, except for *SHOX* on Yp11.2, has been shown to facilitate skeletal growth. *SHOX* encodes a homeoprotein that regulates proliferation, differentiation, and apoptosis of chondrocytes [3]. *SHOX* pathogenic variants are known to cause short stature with or without wrist skeletal malformations [3]. Despite its critical role in skeletal growth, *SHOX* was excluded from the candidates of GCY, because *SHOX* resides within pseudoautosomal region 1 (PAR1) shared by the X and Y chromosomes [3]. Until recently, it was believed that all genes in PAR1 including *SHOX* escape X chromosome inactivation (XCI) and are expressed equally from the X and Y chromosomes in men, and from the active and inactive X (Xa and Xi) chromosomes in women [4].

However, systematic transcriptome analyses conducted by Tukiainen et al. revealed that many PAR1 genes are more strongly expressed in male tissues than in female tissues [5]. Later, our quantitative RT-PCR analyses confirmed male-dominant expression of *SHOX* in cartilage tissues [6]. For example, average relative mRNA levels of *SHOX* against *TBP* in male and female knee cartilage tissues were 0.80 (range, 0–7.3) and 0.056 (0–0.86), respectively. Furthermore, we observed sex-specific methylation of several *SHOX*-flanking CpG sites. DNA methylation levels in intron 2 (a putative enhancer region) negatively correlated with *SHOX* expression levels. Our study, in conjunction with the report by Tukiainen et al. [5], provided the first indication that in cells of female cartilage tissues, incomplete XCI spreading into PAR1 downregulates *SHOX* expression. Considering that the SHOX protein has strong growth-promoting activity, reduced *SHOX* expression in cartilage tissues likely contributes to the relative short stature of women.

In a recent paper published in *Proceedings of the National Academy of Sciences* [7], Berry et al. demonstrated that gene dosage effects of the sex chromosomes are the primary contributors to sex differences in height. The authors analyzed the height and genome data from 928,605 individuals across three large population-based cohorts, including 1225 adults with sex chromosomal aneuploidy. The results suggested that the presence of extra Y and X chromosomes results in height gain of ~8.5 cm and 5.4 cm, respectively, independent of sex hormones, although the results would be compromised by the presence of chromosome imbalance with non-specific growth disadvantage [3]. The contribution of male sex hormones (testicular androgens) was estimated to be ~10.7 cm. Importantly, the authors showed that *SHOX* abnormality exerts a more severe impact on the stature of men than women; a common nonsense variant (R195X) caused height reduction of 18.6 cm and 8.9 cm in men and women, respectively. Based on these data, the authors proposed that the difference in growth-promoting effects between Xi and Y chromosomes (ΔXiY) explains 22.6% of sex differences in height, with *SHOX* being an important factor for ΔXiY. Although the results for the R195X variant seemingly contradict prior observations that wrist skeletal malformations are usually more severe in female patients with *SHOX* abnormalities [3, 8], this discrepancy can be explained by assuming that women are intrinsically prone to chondrocyte dysregulation due to low *SHOX* expression [6]. Alternatively, estrogens may enhance asymmetrical epiphyseal fusion in female patients [3, 8].

**Fig. 1:**
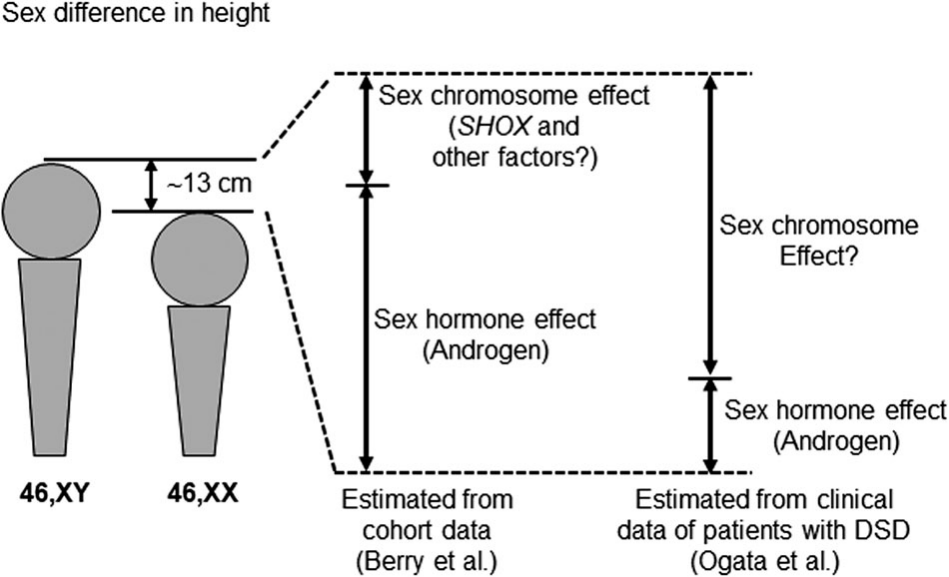
Factors implicated in the sex difference in height. Berry et al. estimated the contribution of sex hormones and sex chromosomes to sexual dimorphism in height from population-based cohort data [7]. The authors assumed that *SHOX* accounts for a certain proportion of the sex chromosome effect. Ogata et al. estimated the effect size of sex hormones and sex chromosomes from the clinical data of patients with disorders of sex development (DSD) [9]

Above-mentioned data indicate that sex-specific epigenetic regulation of *SHOX*, together with testicular androgens, plays a significant role in the height sex difference (Fig. 1). However, the precise effects of *SHOX* and androgens on adult height remain to be clarified. Previous genotype-phenotype correlation analysis for patients with various types of sex hormone disorders, including gonadal dysgenesis, complete androgen insensitivity syndrome, and 46,XX testicular disorders of sex development, have suggested that only ~3.5 cm of sex differences in height can be explained by testicular androgens [9]. This value differs from that reported by Berry et al. (10.7 cm). Moreover, we cannot exclude the possibility that as-yet-unrecognized genes or RNAs on sex chromosomes may exert sex-biased effects on height. Indeed, animal studies have suggested that murine sex chromosomes underlie sexual differences in bone mass and body weight, although mice have no homolog of *SHOX* [10]. Further studies are needed to clarify the precise mechanisms underlying sexual dimorphism in human height.
